# Supplementary figures and images for: “It is because of the love for the job that we are still here”: Mental health and psychosocial support among health care workers affected by attacks in the Northwest and Southwest regions of Cameroon
Source: PLOS Glob Public Health. 2023 Nov 2;3(11):e0002422. doi: 10.1371/journal.pgph.0002422 (PMC10621865; doi:10.1371/journal.pgph.0002422)

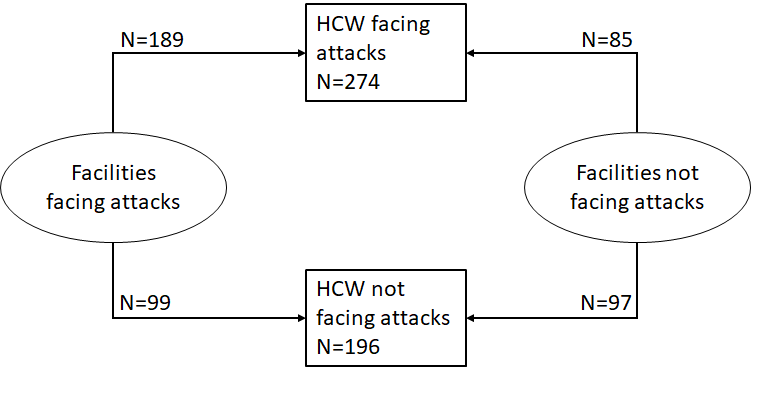

Supplement: S1 Fig — (TIF) [file pgph.0002422.s001.tif]
