# Supplementary material for: “It is because of the love for the job that we are still here”: Mental health and psychosocial support among health care workers affected by attacks in the Northwest and Southwest regions of Cameroon
Source: PLOS Glob Public Health. 2023 Nov 2;3(11):e0002422. doi: 10.1371/journal.pgph.0002422 (PMC10621865; doi:10.1371/journal.pgph.0002422)
Supplement: Attachment — Submitted filename: Ibrahima BARRY REVIEW.docx [file pgph.0002422.s004.docx]

- Lines 51-54: *«Attacks on health care include incidents such as high-impact attacks on health facilities, looting and theft of health assets, obstruction of access to health care, and in particular physical and psychological violence towards health care workers (HCW)».* **Please insert the reference(s) of this sentence.**
- Line90: **in the section** *«General setting – Northwest & Southwest regions of Cameroon»,* **for demographic data I advise you to use national documents (Ministry of Health, National Institute of Statistics) as reference.**
- **In the methodology, in lines 92-93 and 95-96, you have indicated the number of inhabitants in each region. I suggest that you specify the year in question.**
- Line 101*: «MHPSS services in the Northwest and Southwest regions of Cameroon».* **Phrases quoted in this section must be referenced.**
- **RESULTS: I propose to insert a flow chart showing the inclusion process of the participants**
