# Supplementary material for: “It is because of the love for the job that we are still here”: Mental health and psychosocial support among health care workers affected by attacks in the Northwest and Southwest regions of Cameroon
Source: PLOS Glob Public Health. 2023 Nov 2;3(11):e0002422. doi: 10.1371/journal.pgph.0002422 (PMC10621865; doi:10.1371/journal.pgph.0002422)
Supplement: Attachment — Submitted filename: Author reply.doc [file pgph.0002422.s005.doc]

Dear Dr. Tappis,

We would like to thank you and the reviewers for your time and effort in reviewing our manuscript, and for the opportunity you have given us to revise our paper. We have now completed our revisions, and are submitting the edited manuscript. We hope that we have satisfied the reviewers’ concerns, and remain of course available for further clarification or editing if required. Below, please find our point-by-point replies (in bold blue font) to the questions and comments raised by the reviewers.

We would also like to confirm our interest in being included in the “Global Mental Health: challenges, opportunities, and the future of the field” collection.

Warm regards,

Rafael Van den Bergh

For the co-authors

**Reviewer 1**

**Many thanks for your kind feedback on the relevance of our work.**

References need to be included in the following lines: 93 and 96 (population), 103-105, 108. The comparison with other studies in literature in lines 360 and 363 require references as well.

**Thank you for highlighting these omissions – we have now added references to these lines.**

The study relies heavily on self-reported data, which can be unreliable due to recall bias or social desirability bias. Participants may have underreported or overreported their experiences or mental health conditions, which could affect the accuracy of the results. The use of objective measures or independent observers could have helped to validate the self-reported data. I believe the Limitation section should be revised to include this limitation.

**We agree that the reliance on self-reported data is an additional study limitation and have amended the limitations section accordingly.**

HCWs who never experienced attacks may have suffered from secondary trauma, which potentially could have affected their ability to provide care as well for fear of being attacked too. It would have provided more insight on the mental health dynamics on the population sampled as a whole rather than just those affected.

**Thank you for this important comment – indeed, through the design (deliberate selection of 3:2 ratio of attacked vs. non-attacked facilities), the study was not ideally suited to offer a general description of the MH situation of HCW in this context, which was likely compromised through secondary trauma and overall exposure to conflict and displacement. We have adapted the first line of the discussion to better reflect this exclusive focus on the added effect of attacks on healthcare and have added an additional note on this point to the first paragraph of the discussion.**

**Reviewer 2**

Lines 51-54: «Attacks on health care include incidents such as high-impact attacks on health facilities, looting and theft of health assets, obstruction of access to health care, and in particular physical and psychological violence towards health care workers (HCW)». Please insert the reference(s) of this sentence.

**Thank you, we have added a reference to the WHO SSA platform (which lists all types of attacks) to support this statement.**

Line90: in the section «General setting – Northwest & Southwest regions of Cameroon», for demographic data I advise you to use national documents (Ministry of Health, National Institute of Statistics) as reference.

**Thank you – we have now added a reference to the “Populations cibles prioritaires de santé publique” document from the MoH (Health Informations Unit) to support these statements.**

In the methodology, in lines 92-93 and 95-96, you have indicated the number of inhabitants in each region. I suggest that you specify the year in question.

**We have now specified that these figures are based on the 2021 projections of the cited MoH document.**

Line 101: «MHPSS services in the Northwest and Southwest regions of Cameroon». Phrases quoted in this section must be referenced.

**We have now cited the OCHA Humanitarian Response Plan Cameroon, as well as a recent paper by Toguem *et al.* to support these statements.**

RESULTS: I propose to insert a flow chart showing the inclusion process of the participants

**Thank you for this proposal – we have included a brief summary chart describing the participant selection; we have opted to include it as supplemental figure, to avoid cluttering up the main manuscript.**

**Comments in file “correction Landry”**

(Additional comments were found in the pdf file labelled “correction Landry” – we are unsure to which reviewer these should be attributed, and therefore address them here separately)

Congratulations to the team for the spirit of the study. Interesting theme that deals with a reality of public health. If funding is available, to evaluate by a cohort of in-depth study the long-term consequences as well at the level of the targets as on the population and on the health system.

**Thank you for your kind words and for this interesting suggestion. We are in fact planning a dissemination workshop on the findings of this study, which will include the development of future research initiatives, and we will make sure to take this suggestion on board.**

How did you select the districts? It is important to talk about the method used. This is important for the validity of the study.

**We have added a clarification in the Study Populations paragraph of the methods section that the district selection was done in a purposive way, accounting for accessibility and safety of the study teams, and have also adapted the limitations section to indicate that there was no random selection of districts, due to operational constraints.**

Weren't there targets that suffered recent and old attacks? In which category are these last ones classified?

**Thank you for identifying this unclarity in our methods description. Participants were characterised by their most recent attack – this is now clarified in the Data Analysis paragraph of the methods.**
